# Global health equity in United Kingdom university research: a landscape of current policies and practices

**DOI:** 10.1186/s12961-016-0148-6

**Published:** 2016-10-10

**Authors:** Dzintars Gotham, Jonathan Meldrum, Vaitehi Nageshwaran, Christopher Counts, Nina Kumari, Manuel Martin, Ben Beattie, Nathan Post

**Affiliations:** 1 Universities Allied for Essential Medicines - Europe, Berlin, Germany; 2 Faculty of Medicine, Imperial College London, Exhibition Road, London, United Kingdom; 3 Medsin UK, London, United Kingdom; 4 Faculty of Medical Sciences, University College London, Gower Street, London, WC1E 6BT United Kingdom; 5 UCL Institute for Global Health, University College London, 30 Guilford Street, London, WC1N 1EH United Kingdom; 6 Faculty of Medical Sciences, The Medical School, Newcastle University, Framlington Place, Newcastle upon Tyne, NE2 4HH United Kingdom

**Keywords:** Global health, Neglected diseases, Intellectual property, Open access, Research funding, University research

## Abstract

**Background:**

Universities are significant contributors to research and technologies in health; however, the health needs of the world’s poor are historically neglected in research. Medical discoveries are frequently licensed exclusively to one producer, allowing a monopoly and inequitable pricing. Similarly, research is often published in ways that make it inaccessible. Universities can adopt policies and practices to overcome neglect and ensure equitable access to research and its products.

**Methods:**

For 25 United Kingdom universities, data on health research funding were extracted from the top five United Kingdom funders’ databases and coded as research on neglected diseases (NDs) and/or health in low- and lower-middle-income countries (hLLMIC). Data on intellectual property licensing policies and practices and open-access policies were obtained from publicly available sources and by direct contact with universities. Proportions of research articles published as open-access were extracted from PubMed and PubMed Central.

**Results:**

Across United Kingdom universities, the median proportion of 2011–2014 health research funds attributable to ND research was 2.6% and for hLLMIC it was 1.7%. Overall, 79% of all ND funding and 74% of hLLMIC funding were granted to the top four institutions within each category. Seven institutions had policies to ensure that technologies developed from their research are affordable globally. Mostly, universities licensed their inventions to third parties in a way that confers monopoly rights. Fifteen institutions had an institutional open-access publishing policy; three had an institutional open-access publishing fund. The proportion of health-related articles with full-text versions freely available online ranged from 58% to 100% across universities (2012–2013); 23% of articles also had a creative commons CC-BY license.

**Conclusion:**

There is wide variation in the amount of global health research undertaken by United Kingdom universities, with a large proportion of total research funding awarded to a few institutions. To meet a level of research commitment in line with the global burden of disease, most universities should seek to expand their research activity. Most universities do not license their intellectual property in a way that is likely to encourage access in resource-poor settings, and lack policies to do so. The majority of recent research publications are published open-access, but not as gold standard (CC-BY) open-access.

**Electronic supplementary material:**

The online version of this article (doi:10.1186/s12961-016-0148-6) contains supplementary material, which is available to authorized users.

## Background

Universities are significant contributors to the research and development of medicines, and other health products central to global health; for example, a third of innovative medicines registered in the United States of America were developed in universities [1]. In the United Kingdom, universities are important contributors to research globally [2, 3]. In 2013, 26% of all research and development (R&D) in the United Kingdom (by total funding across sectors) was carried out in higher education institutions [4]. The investments in the United Kingdom Research Excellence Framework in 2015 highlights an overall desire for evaluation of research output from United Kingdom universities [5]. In addition, university research will be a crucial component to any strategy to increase R&D in the area of global health, such as that proposed by the Consultative Expert Working Group on Research and Development of WHO [6].

Braveman, writing on equity in global health in 1996, defined equity as “[meaning] *that people’s needs, rather than social privileges, guide the distribution of opportunities for well-being*” [7]. Given that the aim of research is to improve these ‘opportunities for well-being’ in a particular health area, needs-guided distributive decisions are required both in health research funding and in the management of the outputs of research in order for health research systems to be equitable. Accordingly, an equitable system of research and development in health would include (among others) needs-driven research funding that addresses the global burden of disease, commitments to share research findings, and commitments to making the end products of research affordable. To assess the status quo of United Kingdom universities with regard to these facets, we chose to measure research grant distribution as a proxy for research activity, open-access publishing activity to address the sharing of research findings, and the management of intellectual property owing to its close link to end-product affordability.

The Commission on Health Research and Development in 1990 found that only 5% of research spending is on health issues affecting developing countries, where 93% of the disease burden occurred. Reducing this imbalance, they reported, is an “*essential link to equity in development*” [8]. While their recommendations focussed primarily on research capacity strengthening in developing countries, focus in the last decade has expanded to how research policy and institutions in high-income countries could change in order to address this imbalance [6, 9]. Empirically, while poverty-related diseases represent 14% of the global disease burden, worldwide only 1.3% of health R&D expenditure is devoted to this area [10]. In the United Kingdom, a 2015 report by the All-Party Parliamentary Group on Global Health discussed the United Kingdom’s overall contributions to global health and recognised an imbalance between the global burden of diseases and the funding allocated to researching these [3].

The approach universities take to managing intellectual property that results from their research affects the affordability of end products (e.g. medicines, vaccines, diagnostics). Competitive generic production has been shown to achieve rapid price decreases and was instrumental to the global scale-up of HIV treatment [11]. Universities can facilitate equitable access to the fruits of their research by adopting socially responsible licensing (SRL) policies to promote early generic manufacture of medicines, or other mechanisms to promote affordability, in low- and middle-income countries (LMICs) [12]. The WHO Consultative Expert Working Group on Research and Development included SRL (termed ‘equitable licensing’ in their report) in their recommendations for improving the system of research and development to address unmet global needs, and SRL is now regarded as standard practice across universities in North America [12–14]. In the United Kingdom, a recent report by the All-Party Parliamentary Group on Global Tuberculosis recommended that “*SRL should be adopted by academic institutions in the right circumstances*” and “[t]*he UK government should* […] *conduct a study into how SRL could be implemented across* […] *publicly funded research*” [15].

Open-access publication is important in making research accessible globally, particularly in LMICs [16]. The term ‘open access’ is used to describe a range of publishing practices, within which the scope of accessibility varies.^1^ For example, among the academic articles for which full texts are freely available online, only some will have a creative commons CC-BY license – that is, many articles will be copyrighted in ways that restrict their use, reproduction and distribution. The gold standard of open access publishing requires using a CC-BY license [17].

This study measured global health research funding, and research sharing policies and practices of 25 top-funded United Kingdom universities, to provide an overview of current United Kingdom university research practices pertaining to global health.

### The United Kingdom university global health research league table

This study began as a project run in collaboration between two student-run non-profit organisations – Medsin-UK and Universities Allied for Essential Medicines United Kingdom (UAEM UK). Some of the data in this paper have been published as an interactive website at www.globalhealthgrades.org.uk. Data analysis for the online version follows an adapted methodology, available under the Methodology tab.

## Methods

This study included the 25 United Kingdom universities receiving the most Medical Research Council (MRC) funding in 2010–2011 (the most recent year with publicly available data at the time of beginning the study) [18];^2^ 25 was selected as a cut-off for feasibility. Metrics used divide into two sections: research funding and research sharing practices.

### Research funding

For each university, we measured the proportion of total health research funding (THRF) that was attributable to research on two overlapping areas of research, namely neglected diseases (NDs) and health in low- and lower-middle-income countries (hLLMICs). These overlapping categories were used to increase the ability to represent ‘global health’ in our measures, in recognition that ‘global health’ is a broad area of study, which would not be adequately represented by either category alone.

Research on health in low- and lower-middle-income countries (LLMICs) was defined as research for which both (1) the subject of inquiry is primarily in a country, or countries, in the low- or lower-middle-income brackets, and (2) the subject of inquiry is an aspect of human health, defined as belonging to any one or more of the ‘research for health’ categories described by the Global Forum for Health Research, which include biomedical research, research into health policy and systems research, behavioural and social science, and operational research [19]. Countries were classified as low-income or lower-middle-income according to the 2012 World Bank criteria (Additional file 1) [20].

NDs were defined as diseases listed as neglected in the G-FINDER 2011 survey on global ND innovation funding [21]. This list includes HIV/AIDS, malaria, tuberculosis, diarrhoeal diseases, dengue, kinetoplastid and helminth infections, bacterial pneumonia and meningitis, salmonella infections, leprosy, rheumatic fever, trachoma, and Buruli ulcer (Additional file 1). The G-FINDER survey defines diseases as ‘neglected’ based on three criteria; namely, the disease disproportionately affects people in developing countries, there is a need for new products, and there is a market failure in R&D for the disease [21]. To our knowledge, the G-FINDER survey (funded by the Bill & Melinda Gates Foundation) is the only prominent survey of research funding that focuses on NDs [9].

To calculate THRF by university, data on health research grants provided by the five major funders of research in the United Kingdom were collected from the respective funders’ databases, including the United Kingdom Department for International Development (DfID) [22], the European Commission (EC) [23], the Bill & Melinda Gates Foundation [24], MRC [25], and Wellcome Trust [26]. Data were extracted from these databases (links in references) by using their respective websites’ advanced search functions, and downloading results as a spreadsheet where possible or manually transcribing if not. In the online search function, a timeframe of July 1, 2011, to December 31, 2013, was used. Search restrictions of ‘research grant’ and ‘university’ as grant recipient were used for MRC, Bill & Melinda Gates Foundation and Wellcome Trust. EC and DfID have a broader funding remit beyond health, therefore, in order to ensure representative data, not all grants were extracted from the database. For EC, only those grants labelled in the database with a subject related to health, medicine or life sciences were included. For DfID, only those grants classed within the theme of ‘health’ were included. While these five funders do not yield a comprehensive dataset of all health research grants given in the United Kingdom, they are likely to represent the great majority of global health research, representing about 90% of infectious disease research investments in 2010 [27].

Extracted data on research grants were filtered by university, and manually coded as meeting the criteria for research on NDs or hLLMICs, as defined above, or neither. Where multiple collaborator institutions were named on a grant, because the databases used do not state the exact amount awarded to each collaborator, the full amount of the grant was attributed to the ‘lead’ institution. An exception to this was the EC database, where the exact amount awarded to collaborators was available: for EC grants, we counted the amount awarded to the lead institution only. While the areas of research we defined as ‘ND’ or ‘hLLMIC’ overlap, a choice in coding was forced between the two at the discretion of the reviewer. For each university, we calculated the total funding received for research into NDs and hLLMICs, and the proportion this funding represented of total health research funding received from the five funders given above.

Primary data collection and coding of all extracted grant descriptions were performed independently by two reviewers, each blinded to the selection of the other. Coding for each search result was compared and disagreements resolved by consensus.

No adjustments were made for inflation. EC and Gates Foundation grants, reported in the databases in EUR and USD, respectively, were converted to GBP using the average exchange rate over 2011–2014 [28].^3^

### Research sharing: patents and open access

This section assessed university patenting and licensing of health-related technologies, and open access publication practices. ‘Technology transfer office’ (TTO) is a generic term used to describe the office or entity within a higher education institution that manages intellectual property associated with the institution. ‘Health-related technologies’ include, for example, medicines, vaccines and diagnostics. Data were collected by self-reporting by TTOs or their equivalent, via responses to an online questionnaire and/or a request for information made under the United Kingdom Freedom of Information Act 2000 (FOI) (Additional file 2).

TTOs were first sent an online questionnaire by email with reminders sent a minimum of four times by email and two times by telephone over a 12-week period beginning July 6, 2014. FOI requests were then sent to universities that did not respond fully by the end of the 12-week period, seeking the same information as the online survey. We designed FOI requests in recognition of the fact that our requested data could be held by TTOs in a variety of formats – the wording of the FOI request was thus slightly different to the survey. The FOI requests also contained an invitation to complete the original online survey, thus removing the need for providing information through the FOI process.

The following data were extracted from responses:

- The percentage of the university’s health technology licensing contracts signed in the last year that were non-exclusive
- The percentage of all health technologies in the past year for which patents were sought in LMICs^4^
- The percentage of the university’s health technology licensing contracts signed in the past year that included provisions to promote access to the technologies in LMICs (this was assessed from survey responses only)

Systematic searches of university websites and public databases were also conducted so that, where possible, findings could be validated by more than one source. Primary data collection and coding was performed independently by at least two researchers, each blinded to the coding of the other. Where coding differed, a decision was reached by consensus.

To assess university commitment to SRL, we systematically searched the university’s website using the website’s search engine, supplemented by a similar search in the Google search engine, using the following terms: “[university name]”, “global access licensing”, “socially responsible licensing”, “equitable access licensing”, “access to medicines”, “university licensing”, “technology transfer”. We supplemented this search by reviewing the list of signatories to Stanford University’s ‘Nine Points to Consider in Licensing University Technology’ and the ‘Statement of Principles for the Equitable Dissemination of Medical Technologies’ [14, 29], and by the inclusion of a relevant question in the survey mentioned above (question 2, Additional file 2). Statistical correlation was calculated for the presence of an SRL policy versus the percentage of licenses that were non-exclusive, as well as for the percentage of licenses containing access provisions versus percentage that were non-exclusive, using Somers’ D test.

University commitments to promoting open-access publication were assessed by two independent reviews of the page, if one existed, of the university website, that outlined their policy on open access publication. We supplemented this search by reviewing the list of signatories to the Compact for Open-Access Publishing Equity [30] and the list of institutions listed in the Registry of Open Access Repositories Mandatory Archiving Policies [31]. These databases provide lists of universities that have open access publication funds, and an institutional open-access publishing mandate and/or policy, respectively. We measured the proportion of research articles published as ‘free-access’, which signifies academic publications for which full-text versions are freely available online, but might have limited re-use rights [32], and the proportion published as CC-BY.

We calculated the percentage of each university’s health-related research output for which the full text is freely available online within 1 year of publication (free access). Percentages were calculated by dividing the number of citations affiliated to a university in PubMed Central (PMC) by the number of citations affiliated to it in PubMed. PMC indexes only publications for which full-text versions are freely available, while PubMed indexes publications without this restriction. PMC results can, in practice, be considered a subset of PubMed that are freely available as full-text versions [33].

We used the following filters: “type – journal article” (PubMed only); “affiliation – (university name)”; “publication date: 1/8/20121/8/2013”. 5 A second search was done using a “cc-by license” filter in PMC to determine the proportion of articles in the PMC subset that used a CC-BY license.

### Statistical analysis

Results with a value greater than the upper quartile plus 1.5 times the interquartile range were considered outliers. Statistical tests were run on data from which outliers had been removed; for ND-attributable funding, London School of Hygiene and Tropical Medicine (LSHTM) and the University of Reading were excluded; for hLLMIC-attributable funding, LSHTM and the University of Leeds were excluded (Table 1). Spearman’s rank correlation coefficient (*r*) was used to test correlations of proportional ND-/hLLMIC-attributable funding to THRF (Table 1), to test correlations between ND-/hLLMIC-attributable funding and licensing and open-access publishing practices (Additional file 3), and to test correlations between THRF and publishing practices. Somers’ D test (rank-biserial) was used to assess correlations given in Table 3. All statistical tests were run using Small Stata software version 10.0, except Somers’ D, which was calculated using SPSS version 21.

**Table 1:** Funding for research on neglected diseases and health in low- and lower-middle-income countries

University	Funding attributable to research on neglected diseases, £ (percentage of THRF in brackets)	Funding attributable to research on health in LLMICs, £ (percentage of THRF in brackets)	Total health research funding in 2011–2014, £ (THRF)
Cardiff University	0 (0.0)	126,286 (0.3)	48,883,350
Imperial College	17,826,708 (11.0)^a^	5,542,297 (3.40)	162,093,401^a^
King’s College London	2,676,930 (2.4)	6,205 (0.0)	110,854,979
LSHTM	24,383,502 (32.5)^a^	22,883,802 (30.5)^a^	74,970,446
Newcastle University	0 (0.0)	517,735 (0.9)	59,051,690
Queen Mary	625,257 (2.7)	584,374 (2.5)	23,404,007
University College London	6,321,359 (2.6)^a^	7,895,999 (3.2)^a^	245,303,666^a^
U. of Aberdeen	0 (0.0)	507,154 (2.4)	20,816,124
U. of Birmingham	2,808,451 (8.0)	1,464,820 (4.2)	35,067,638
U. of Bristol	589,409 (1.3)	0 (0.0)	44,036,199
U. of Cambridge	688,439 (0.3)	3,085,230 (1.2)	260,465,308^a^
U. of Dundee	5,980,568 (7.2)	301,480 (0.4)	82,629,476
U. of Edinburgh	4,744,693 (3.8)	3,041,556 (2.4)	125,460,071
U. of Glasgow	1,632,483 (3.9)	0 (0.0)	41,556,952
U. of Leeds^b^	0 (0.0)	7,500,000 (23.3)^a^	32,221,557
U. of Leicester	4,000 (0.0)	0 (0.0)	16,756,360
U. of Liverpool	3,540,839 (10.8)	756,015 (2.3)	32,858,179
U. of Manchester	440,484 (0.6)	0 (0.0)	69,608,229
U. of Nottingham	673,835 (3.1)	366,123 (1.7)	21,946,156
U. of Oxford	51,697,856 (17.6)^a^	14,028,309 (4.8)^a^	293,780,277^a^
U. of Reading	1,130,489 (19.6)	0 (0.0)	5,778,531
U. of Sheffield	0 (0.0)	0 (0.0)	28,309,890
U. of Southampton	0 (0.0)	548,557 (6.8)	8,056,867
U. of Sussex	1,558,982 (4.0)	111,824 (0.3)	39,160,020
U. of Warwick	299,060 (1.7)	1,477,190 (8.3)	17,810,933
Total	127,623,343 (6.71)	70,744,957 (3.72)	1,900,880,305
Median proportion across institutions	2.6 (IQR 7.2)	1.7 (IQR 3.4)	N/A
Share of total within-column funding granted to top four institutions	79%	74%	51%
Correlation with THRF	0.390 (*P* = 0.066)	0.074 (*P* = 0.736)	N/A

*LLMICs* low- and lower-middle-income countries, *LSHTM* London School of Hygiene and Tropical Medicine, *IQR* inter-quartile range, *THRF* total health research funding

^a^Values for top four institutions by absolute funding within each column

^b^All hLLMIC-attributed funding at the University of Leeds (£7.5 million) represents a single grant awarded by DfID

## Results

### Research funding

**Fig. 1:**
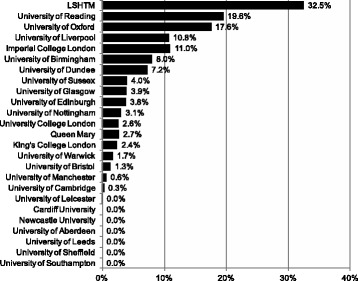
Proportion of total health research funding attributable to research on neglected diseases in 2011–2014

**Fig. 2:**
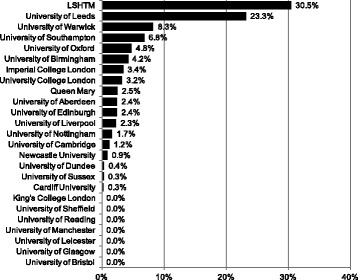
Proportion of total health research funding attributable to research on health in low- and lower-middle-income countries in 2011–2014

For the 3-year period 2011–2014, we identified a THRF of £1.9 billion granted to the 25 institutions included in this study, of which £128 million were to research on NDs and £71 million were to research on hLLMICs, equivalent to 6.7% and 3.7% of THRF, respectively. Across institutions, the median proportion of THRF attributable to NDs and hLLMICs was 2.6% (IQR 7.2%) and 1.7% (IQR 3.4%), respectively (Table 1). Proportional ND/hLLMICs research funding for individual universities is shown in Figures 1 and 2, respectively. Proportional funding for research on NDs or hLLMICs did not correlate significantly with the THRF of the institution (NDs: *r* = 0.390, *P* = 0.066; hLLMICs: *r* = 0.074, *P* = 0.736).

The top four institutions by absolute funding (indicated in Table 1) were responsible for 79% and 74% of total ND and total hLLMIC research funding granted to all 25 institutions, respectively. The top four by absolute funding were not the same as the top four by proportional funding. For the time frame 2011–2014, we found that six universities had no ND-attributable research funding, and six universities had no hLLMIC-attributable research funding.

There was significant variability between relative contributions by funders to THRF, ND-attributable, and hLLMIC-attributable research funding (Additional file 4). Wellcome Trust and the MRC were responsible for far larger proportions of funding of United Kingdom university health research, in general, than the EC, DfID, or the Gates Foundation. In funding for ND research, the Gates Foundation contributed nearly as much as the Wellcome Trust and the MRC, while EC and DfID contributions remained lower. In funding for hLLMICs research, DfID contributed the greatest amount, with slightly lower contributions by Gates Foundation, MRC and the Wellcome Trust. Funding contributions were more even between funders in hLLMIC research funding than they were in total health research funding or ND research funding.

Three-year trends are shown in Additional file 4. Trends in THRF show mixed year-on-year funding changes. The University of Oxford and the University of Newcastle show an upward trend, while King’s College London and LSHTM show a downwards trend. All other universities show unclear trends over 3 years. In ND research funding, upwards funding trends were seen for the University of Oxford, Imperial College London, University College London (UCL), the University of Edinburgh, the University of Birmingham, and the University of Glasgow. LSHTM showed a downwards trend. In hLLMICs research, trends were unclear, except for LSHTM, which showed a downwards trend.

### Licensing of patented technologies

Seven of the 25 universities had public commitments to making the products of their research affordable in developing countries through SRL policies (Table 2). In the 20 universities that licensed health technologies within the last year, approximately 30% of all licenses were non-exclusive. Four universities reported licensing 81–100% of licenses non-exclusively, while 11 reported licensing 0–20% non-exclusively (Table 3). Two universities reported including access provisions in 81–100% of licenses, whereas 19 reported including these in 0–20% of licenses (Table 2).

**Table 2:** Overview of intellectual property licensing policy landscape

University	Has publicly committed to general principles of SRL	Plans to endorse SRL within 1 year	Has endorsed specific licensing strategies for promoting access	Has endorsed licensing strategies that prioritise generic production
University College London	Yes	N/A	Yes	No
University of Edinburgh	Yes	N/A	Yes	No
University of Manchester	Yes	N/A	Yes	No
University of Oxford	Yes	N/A	No	No
Imperial College London	Yes	N/A	No	No
University of Dundee	Yes	N/A	No	No
University of Bristol	Yes	N/A	No	No
LSHTM	No	Yes	No	No
University of Liverpool^a^	No	Yes	No	No
University of Aberdeen	No^b^	No	No	No
University of Nottingham	No^b^	No	No	No
University of Sussex	No^b^	No	No	No
University of Birmingham	No	No	No	No
University of Cambridge	No	No	No	No
Cardiff University	No	No	No	No
University of Glasgow	No	No	No	No
King’s College London	No	No	No	No
University of Leeds	No	No	No	No
University of Leicester	No	No	No	No
Newcastle University	No	No	No	No
Queen Mary	No	No	No	No
University of Reading	No	No	No	No
University of Sheffield	No	No	No	No
University of Southampton	No	No	No	No
University of Warwick	No	No	No	No

Data from systematic website searches (policies only), self-reporting via electronic survey, and responses to requests for information made under the Freedom of Information Act 2000 (practices only)

*SRL* socially responsible licensing, *LSHTM* London School of Hygiene and Tropical Medicine

^a^Policies on intellectual property management in effect at the University of Liverpool do not affect the management of intellectual property at the Liverpool School of Tropical Medicine, the latter of which is not included in this study

^b^Responses to surveys indicated general commitment to principles of SRL by the technology transfer office, but this commitment was not official or public at the time of data collection

**Table 3:** Overview of licensing practices (2012–2013)

University	Publicly available SRL policy	Proportion of licenses that were non-exclusive	Proportion of licenses that included provisions to promote access in LLMICs
University of Bristol	Yes	96%	44%
Newcastle University	No	90%	–
University of Leeds	No	82%	–
University of Oxford	Yes	71–100%	81–100%
University College London	Yes	51–70%	41–60%
University of Glasgow	No	51–70%	0–20%
University of Edinburgh	Yes	31–50%	0%
Imperial College London	Yes	31–50%	21–40%
University of Leicester	No	11–30%	0–20%
University of Sheffield	No	11–30%	0–20%
University of Aberdeen	No	0–10%	81–100%
University of Birmingham	No	0–10%	0–20%
University of Manchester	Yes	0–10%	0–20%
University of Sussex	No	0–10%	0–20%
University of Southampton	No	9%	–
University of Dundee	Yes	0%	–
King's College London	No	0%	–
University of Nottingham	No	0%	–
Queen Mary	No	0%	–
University of Warwick	No	0%	–
Cardiff University	No	N/A	N/A
University of Liverpool	No	N/A	–
LSHTM	No	N/A	N/A
University of Reading	No	N/A	N/A
University of Cambridge	No	^a^	–
Correlation with presence of SRL policy	–	0.469 (*P* = 0.051)	0.286 (*P* = 0.346)

Correlations given as Somers’ D, calculated from datasets with ‘N/A’,’—‘, and ‘^a^’ values censored

‘N/A’ indicates no licenses were executed during the time period

‘–’ indicates that this question was not answered via electronic survey, and could not be ascertained otherwise

*SRL* socially responsible licensing, *LSHTM* London School of Hygiene and Tropical Medicine, *LLMICs* low- and lower-middle-income countries

^a^Refused to provide information requested under the Freedom of Information Act for this question

The presence of an SRL policy at a university positively predicted the level of non-exclusive licenses as well as the use of access provisions in exclusive licenses, though neither of these relationships was statistically significant (Table 3). In general, levels of patent seeking in LLMICs and BRICS countries were very low. It was more common for patents to be sought in BRICS countries than in other LLMICs (Additional file 3).

Neither absolute nor proportional ND or hLLMIC funding showed statistically significant correlation to licensing practices (Additional file 3).

### Open access publishing

We differentiate between institutional open-access publishing funds – funds offered by the university to cover open-access publication fees (article processing charges) – and open-access publishing funds offered to researchers by extra-institutional bodies such as the Wellcome Trust’s Charity Open Access Fund and Research Councils UK’s open access block grants. All 25 universities included in the study were recipients of Charity Open Access Fund and/or Research Councils UK open-access publishing funds [34, 35]. Institutional funds, however, were found in only three institutions (Table 4). Institutional open access policies were found in 15 universities (Table 4).

**Table 4:** Open access policies and funds

University	Institutional open access policy	Institutional open access publishing fund^a^
Imperial College London	+	+
Newcastle University	+	+
University of Aberdeen	+	
University of Bristol	+	
University of Cambridge	+	
University College London	+	
University of Dundee	+	
University of Edinburgh	+	
King’s College London	+	
University of Leeds	+	
University of Leicester	+	
University of Nottingham	+	
University of Oxford	+	
University of Southampton	+	
University of Warwick	+	
LSHTM		+
University of Birmingham		
Cardiff University		
University of Glasgow		
University of Liverpool		
University of Manchester		
Queen Mary University		
University of Reading		
University of Sheffield		
University of Sussex		

*LSHTM* London School of Hygiene and Tropical Medicine

^a^Funds other than those provided by the Wellcome Trust/Charity Open Access Fund or Research Councils UK. All 25 universities included in the study were recipients of Charity Open Access Fund and/or Research Councils UK open-access publishing funds

**Fig. 3:**
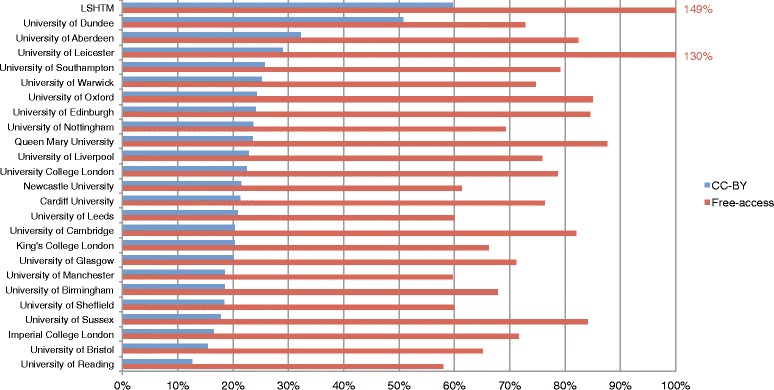
Free-access and CC-BY publications as percentage of total health-related publications, 2012–2013

Of health-related research articles published by the universities surveyed in 2012–2013, 76% had full texts freely available online. The proportion of individual universities’ total health-related publications that were free-access in 2012–2013 ranged from 58% to 100%, with a slight negative skew and a median of 75% (IQR, 16%); 23% (IQR, 7%) of publications were free-access and had a CC-BY license, which allows for unrestricted distribution and re-use of content. Figure 3 shows the proportion of total journal articles published in 2012–2013 published as free-access and CC-BY for each university.

No correlation was found between THRF and proportion published as free-access (*r* = 0.287, *P* = 0.164) or between THRF and proportion published as CC-BY (*r* = 0.034, *P* = 0.873). The percentage of articles published as free-access and percentage published as CC-BY were statistically correlated (*r* = 0.643, *P* = 0.0005). Proportional hLLMIC research funding was moderately correlated to both proportion of articles published as free-access (*r* = 0.588, *P* = 0.004) and CC-BY (*r* = 0.491, *P* = 0.02) (Additional file 3). Absolute hLLMIC funding, and absolute and proportional ND funding were not significantly correlated to open-access publishing practices.

There were two institutions (LSHTM, Leicester) for which the number of publications in PMC exceeds that in PubMed, resulting in a free-access percentage of greater than 100%. This is likely a result of the slight differences in the types of literature that are included in the two different databases [33]. Advanced search functions also differ slightly. Consequently, while the percentage values presented in Fig. 3 can be used for the comparison of universities, their interpretation as absolute measures of publication practices is limited at this stage.

## Discussion

A number of trends have been demonstrated: a significant gap in funding for global health research, disparities between institutions’ level of commitment, and irregular adoption of research sharing policies and practices by United Kingdom universities.

The low median proportion of funding given to LLMIC health and ND research shows that most universities are carrying out less research into these areas than would be expected if funding of research were equivalent to the proportion of global burden of disease attributable to these categories. The median proportion of total health research funding devoted to NDs at United Kingdom universities (2.6%) is approximately five times lower than the global burden of NDs, where NDs are defined by the G-FINDER list (13.8% of the global burden of disease) [10, 21]. Overall ND funding identified totalled 6.7% of all health research funding identified, that is, about half the global burden of disease. For three universities – LSHTM, the University of Reading and the University of Oxford – the proportion of total research funding for research on NDs exceeded the 13.8% threshold (Fig. 1). In terms of ‘equity’ as fulfilled by distributive decisions based on need, this translates to United Kingdom university research being ‘halfway’ to a proportional distribution of ND research funding that could be called equitable. Indeed, it can be argued that, for an equitable funding distribution to be reached, considering historical underfunding, ND research funding would, for a period of time, need to be significantly greater than the corresponding global burden of disease in order to ‘catch up’.

The median proportion of research funding devoted to hLLMIC research at United Kingdom universities is 1.7% and overall United Kingdom hLLMIC funding of all health funding is 3.7%, which is 1.5 and 1.8 times less than for ND research, respectively. LLMICs account for 59% of the global burden of disease (measured in disease-adjusted life years; authors’ own calculation using http://vizhub.healthdata.org/gbd-compare/). The category of ‘research on hLLMICs’ is not as well-established as that of NDs; our definition was based on the grant specifically mentioning a focus on one or more developing countries. It is likely that, in many cases, research on health issues affecting mainly developing countries may be described in the grant only in terms of the health issue itself and not its geographical relevance. Our findings regarding hLLMIC research funding are thus meant to complement findings on NDs, although they cannot be directly comparable to figures for global burden of disease in the way that ND figures can.

The top four institutions by absolute funding represent 79% of total ND research funding and 74% of total hLLMIC research funding. This inter-institutional inequality is greater than inter-institutional inequality in total health research funding, where the top four institutions by absolute funding received 51% of all funding. The notable concentration of neglected disease and LLMIC health research funding in a few universities has a number of possible explanations. It may be that these areas of work are tacitly considered specialist areas – and thus a few specialist centres emerge. Alternatively, it may be that these academic areas – as areas of historical neglect – require ‘confidence’ to engage in, that is, an institution has to be relatively large and well-funded in order to enter these areas of research that have historically been less prestigious, and traditionally considered less profitable. Three-year funding trends reflect both the dominance of a small number of institutions in both ND and hLLMIC research, as well as the greater inter-institutional inequality in these areas than in THRF (Additional file 4). The degree of inter-institutional inequality did not seem to change across the 3 years in THRF, ND or hLLMIC funding, though this was not statistically tested. The trends also demonstrate irregularity in funding – this may be due to the commonality of a low number of individual multi-million grants (as opposed to a larger number of smaller grants). Our methodology has counted the grant in the year it was awarded (or the start year for the project, depending on the database), instead of spreading the grant money over the whole time of the project. Although this is a limitation of time-trend analysis, we do not see this as a significant limitation for the main aim of this analysis – to provide a ‘snapshot’ overview of funding.

Analysis of proportional funding – ND/hLLMIC funding as a proportion of the institution’s THRF – paints a different picture to absolute ND/hLLMIC funding: many universities with extensive total health research funding rank relatively low when universities are ordered by proportional funding. For example, only 0.3% of research funding at the University of Cambridge (THRF £260 million) were attributable to ND research; at UCL (THRF £245 million), only 2.6% were attributable to ND research – placing both multiple ranks below universities with far smaller THRF, such as the Universities of Birmingham, Dundee and Reading (Fig. 1). Similarly, in hLLMIC research, the Universities of Leeds, Warwick and Southampton rank numerous places above Edinburgh and Cambridge (Fig. 2). In some cases, this is due to a comparatively small number of large grants summated representing a significant portion of a smaller universities’ THRF.^6^

In general, the high variability of proportional funding across leading universities in the United Kingdom points to a lack of consistent commitment to these research areas (Figs. 1 and 2). The finding that current work is concentrated in the top institutions may inform a policy debate on whether to support this trend or promote a ‘standard minimum threshold’ of research into neglected areas at all institutions. There are numerous examples of smaller and less well-funded universities that are ‘pulling more than their weight’, committing to a neglected research area, and simultaneously, numerous examples of large institutions with comparatively little commitment to global health research. A discrepancy between the funding of research on individual neglected tropical diseases and their relative disease burdens has also been described [2].

Following Braveman’s framework, equity requires focussed, systemic changes in resource allocation. Systemic change in United Kingdom research could manifest as either commitment to a (higher) median level of ND and hLLMIC research investment across institutions, or intentional ‘delegation’ of the work to a smaller number of institutions: either approach would be consistent with progress towards equity. It is likely that, while the ‘higher median’ approach could come primarily from decisions taken by individual universities, a ‘delegation’ approach to systemic change would have to come from decisions by funders. Nothing precludes both approaches from being taken simultaneously. Funders and researchers are interdependent in setting the research agenda: funders cannot fund research in areas where there are no researchers, and researchers cannot do research in areas where there is no funding.

Overall, investments in ND and hLLMIC research in the United Kingdom are at less than half the levels that would expected to match global disease burden. To overcome this mismatch, there is a need for funders of United Kingdom university research to prioritise neglected areas of research in the grant programmes they offer, and for universities to prioritise this research internally.

Monopolies permitted by patent rights have led to prices that exclude the developing world from access, for example, in HIV/AIDS, hepatitis C, and cancer medicines [36–38]. That this is inequitable is obvious, as there is no lack of need for these medicines in poorer countries. Responsible management by universities of the intellectual property deriving from their research can be an important ‘up-stream’ intervention. Policies that safeguard end-product affordability in university agreements on the licensing of intellectual property rights – socially responsible licensing (SRL) policies – have had demonstrated successes in Canada and the United States [39, 40].

The low prevalence of SRL policies (Table 2) among United Kingdom universities likely reflects lack of awareness of these approaches in the United Kingdom. In the United States, most top universities have signed statements committing to SRL [12–14]; United Kingdom universities appear to be lagging behind their United States counterparts. No correlation was found between proportional ND or hLLMIC funding and SRL licensing practices (Additional file 3). Nevertheless, three out of the top four universities by funding for ND research (Table 1) have publicly available SRL policies, and the fourth of those (LSHTM) has reported plans to endorse SRL within 1 year. One explanation may be that universities that conduct more ND research are more aware of developments in policy related to treatment access such as SRL. Another factor may be that these four universities are relatively highly funded and have larger TTOs, and are therefore more likely to be aware of newer policy trends, than most others.

As these provisions are conceptually new, the two universities reporting 81–100% of licenses containing access provisions are of particular interest (University of Aberdeen; University of Oxford). Case studies of practices at these universities would offer useful insight into the wider United Kingdom implementation, in line with the recent recommendations of the All-Party Parliamentary Group on Global Tuberculosis [15]. The lack of statistical significance of the positive correlations found between the presence of an SRL policy and both non-exclusive licensing and access provisions (Table 3) could be due to the relatively low number of universities that have adopted such a policy. The technology transfer office at UCL has indicated that the adoption of an SRL policy increased their ability to negotiate inclusion of provisions that promote affordability in licensing agreements [41].

Where SRL policies have been adopted, transparency and accountability mechanisms in the implementation of these policies remain important [13]. Further research on the eventual fate of health products developed in United Kingdom universities would be valuable, for example, assessing their availability in resource-poor settings. In the context of established North American precedents, and endorsement by the Consultative Expert Working Group and other bodies, we expect both universities and funders of research in the United Kingdom to develop plans for implementing SRL policies more widely in the near future.

The proportion of health-related research articles published as free-access (76%) is similar to reported average European rates [42]. The low variability (IQR 16%) between universities shows that open-access adoption is fairly uniform, likely due to effective advocacy for policy adoption and increasing requirements for, and support of, open-access publication by funders. Increasingly, funders also require publishing with a CC-BY license [43]. CC-BY licensing, allowing free reuse of the published material with no restrictions other than a requirement of appropriate attribution, is considered superior to free-access publication alone [17]. In this regard, there is a considerable gap in proportion published as free-access versus CC-BY – 76% versus 23%, respectively. The top four institutions by THRF – Oxford, Cambridge, UCL, and Imperial – are not, overall, highly ranked in proportional CC-BY publications, spread over a range of 16% to 24% (Fig. 3), and we found no correlation between total research funding and free-access or CC-BY publishing. In the United Kingdom, the adoption of open-access publishing in health research is not being led by larger institutions.

The finding that proportional hLLMIC research funding was significantly correlated to both proportional free-access and CC-BY publishing (r = 0.588, *P* = 0.004 and r = 0.491, *P* = 0.02) merits further investigation. A potential causal mechanism may be that funders that preferentially fund hLLMIC research have stronger open-access policies attached to grants. The interactions between funders and open-access publishing are, however, beyond the scope of this study.

In Braveman’s equity framework, open-access publishing and SRL policies contribute to an equitable health research system by both ‘sharing progress’ and by ‘levelling up’ – bringing everyone to the highest standard, instead of reducing standards for those experiencing the best opportunities.

Our measures of global health research and research sharing have limitations. Financial expenditure is an imperfect measure of research output. This study may not have captured all global health work being undertaken at the universities surveyed due to limitations in time frame, the level of detail provided in databases and the use of proxy definitions for ‘global health’ (ND and hLLMIC). The attribution of full grant amount to the lead institution, in cases where multiple collaborators were named, has the potential bias of rewarding larger institutions with larger administrative capacity, and thus skewing the distribution towards larger institutions (except for EC grants, where it was possible to separate the amount to lead institution).

Data based on responses to surveys and FOI requests must be treated with caution due to potentially variable interpretation of certain terms; in particular, ‘provisions that promoted access’ were not defined and respondents were asked to respond based on what their understanding of the phrase was. This study did not conduct detailed analysis of university policies on open-access publishing and intellectual property management. As both types of policies can vary greatly in breadth, strength and specific mechanisms mandated, further analysis of these policies is eagerly awaited.

## Conclusion

Global health research in United Kingdom universities is concentrated in a small number of institutions and is generally underfunded as an area of health research, considering the global burden of disease that it represents. NDs represent 13.8% of the global burden of disease but only 6.7% of all health research funding [10]. Between individual universities, the median proportion of THRF attributable to ND research (2.6%) was five times below what would be expected given the proportion of global disease burden they represent (13.8%) [10]. For research on hLLMICs, both proportional indicators were even lower (proportion of THRF – 3.7%, median proportion between universities – 1.7%). Most universities lack socially responsible licensing policies (18 of 25), and do not license their intellectual property in a way that is likely to encourage access in resource-poor settings. The majority of research publications (77%) are not licensed with gold-standard open access, despite most universities (15 of 25) having open access policies in place. To meet a globally equitable level of global health research, where research funding distribution is commensurate with the global burden of disease, funders and universities would need to expand their research activity in global health.

Advocacy is needed to promote the adoption of SRL policies at United Kingdom universities, with subsequent follow-up on effective implementation. While open-access policies are widespread, CC-BY open-access publication is still far from the norm. For a health research system to be equitable, progress must be shared by allowing everyone to enjoy the highest possible standard: to stand on the shoulders of giants. To this end, there is a need for increased commitments among United Kingdom universities to policies that safeguard the affordability of healthcare products and the accessibility of research papers.

## Additional files

Additional file 1: 2012 World bank country classification and list of neglected diseases. (DOCX 118 kb)
Additional file 2: Copy of online survey and FOI request letter. (DOCX 877 kb)
Additional file 3: Funding breakdown by funder, and three-year funding trends. (DOCX 80 kb)
Additional file 4: Patenting activity in BRICS countries and LLMICs by university, and additional inter-metric correlation statistics. (DOCX 594 kb)

## Abbreviations

DfID: United Kingdom Department for International Development
EC: European Commission
FOI: Freedom of Information Act 2000
hLLMIC: Health in low- and lower-middle-income countries
IQR: Interquartile range
LMICs: Low- and middle-income countries
LSHTM: London School of Hygiene and Tropical Medicine
ND: Neglected disease(s)
PMC: PubMed Central
R&D: Research and development
SRL: Socially responsible licensing
THRF: Total health research funding
TTO: Technology transfer office
UCL: University College London

## Footnotes

1 The Budapest Open Access Initiative defines open access thus: “*By ‘open access’ to* [peer-reviewed research literature]*, we mean its free availability on the public internet, permitting any users to read, download, copy, distribute, print, search, or link to the full texts of these articles, crawl them for indexing, pass them as data to software, or use them for any other lawful purpose, without financial, legal, or technical barriers other than those inseparable from gaining access to the internet itself. The only constraint on reproduction and distribution, and the only role for copyright in this domain, should be to give authors control over the integrity of their work and the right to be properly acknowledged and cited*” [17].

2 Although it was one of the top 25 recipients of MRC funding 2010–2011, the Babraham Institute was excluded due to not being a university, despite offering post-graduate programmes as a partner of the University of Cambridge. Further, the authors recognise the Liverpool School of Tropical Medicine as a major contributor to global health and neglected disease research in the United Kingdom, although it does not rank within the top 25 United Kingdom universities by MRC funding in 2010–2011.

3 1 EUR = 0.842745667 GBP; 1 USD = 0.631429333 GBP.

4 Patent Cooperation Treaty applications that had not yet matured into national and/or regional applications were excluded from calculations of the percentage.

5 A date range of 12–24 months prior to the data collection date was adopted to avoid missing PMC-archived articles under a full-text embargo period of up to 1 year.

6 For the University of Leeds, a single £7.5 million grant (for the COMDIS-HSD consortium, http://comdis-hsd.leeds.ac.uk/) for hLLMIC research represented 23.3% of their total health research funding in 2011–2014.
