# Supplementary material for: Global health equity in United Kingdom university research: a landscape of current policies and practices
Source: Health Res Policy Syst. 2016 Oct 10;14:76. doi: 10.1186/s12961-016-0148-6 (PMC5057402; doi:10.1186/s12961-016-0148-6)
Supplement: Additional file 1: — 2012 World bank country classification and list of neglected diseases. (DOCX 118 kb) [file 12961_2016_148_MOESM1_ESM.docx]

| Lower-middle-income countries | | Low-income Countries | |
| --- | --- | --- | --- |
| Armenia | Mongolia | Afghanistan | Myanmar |
| Bhutan | Morocco | Bangladesh | Nepal |
| Bolivia | Nicaragua | Benin | Niger |
| Cabo Verde | Nigeria | Burkina Faso | Rwanda |
| Cameroon | Pakistan | Burundi | Sierra Leone |
| Congo, Rep. | Papua New Guinea | Cambodia | Somalia |
| Côte d'Ivoire | Paraguay | Central African Republic | South Sudan |
| Djibouti | Philippines | Chad | Tajikistan |
| Egypt, Arab Rep. | Samoa | Comoros | Tanzania |
| El Salvador | São Tomé and Principe | Congo, Dem. Rep. | Togo |
| Georgia | Senegal | Eritrea | Uganda |
| Ghana | Solomon Islands | Ethiopia | Zimbabwe |
| Guatemala | Sri Lanka | Gambia, The |  |
| Guyana | Sudan | Guinea |  |
| Honduras | Swaziland | Guinea-Bissau |  |
| India | Syrian Arab Republic | Haiti |  |
| Indonesia | Timor-Leste | Kenya |  |
| Kiribati | Ukraine | Korea, Dem. Rep. |  |
| Kosovo | Uzbekistan | Kyrgyz Republic |  |
| Lao PDR | Vanuatu | Liberia |  |
| Lesotho | Vietnam | Madagascar |  |
| Mauritania | West Bank and Gaza | Malawi |  |
| Micronesia, Fed. Sts. | Yemen, Rep. | Mali |  |
| Moldova | Zambia | Mozambique |  |
| 2012 World Bank country classifications. Historical income classifications available from http://siteresources.worldbank.org/DATASTATISTICS/Resources/OGHIST.xls | | | |

| G-FINDER 2011 diseases |  |
| --- | --- |
| HIV/AIDS | **Helminth infections** |
| Malaria (any strain) | Roundworm (ascariasis) |
| Tuberculosis | Hookworm |
| Diarrhoeal diseases | Whipworm (trichuriasis) |
| Rotavirus | Strongyloidiasis & other intestinal roundworms |
| Enterotoxigenic *E. coli* (ETEC) | Lymphatic filariasis (elephantiasis) |
| Cholera | Onchocerciasis (river blindness) |
| Shigella | Schistosomiasis (bilharziasis) |
| *Cryptosporidium* | Tapeworm (cysticercosis/taeniasis) |
| Enteroaggregative *E. coli* (EAggEC) | **Bacterial pneumonia & meningitis** |
| Giardia | *Streptococcus pneumoniae* |
| Dengue | *Neisseria meningitides* |
| Kinetoplastids | **Salmonella infections** |
| Chagas’ disease | Non-typhoidal *Salmonella enteric* (NTS) |
| Leishmaniasis | Typhoid and paratyphoid fever (*S. typhi, S. paratyphi A)* |
| Sleeping sickness | **Trachoma** |
| Leprosy | **Buruli ulcer** |
| Rheumatic fever |  |
| List from Moran M, Guzman J, Abela-Oversteegen L, Liyanage R, Omune DB, Wu L, et al. Neglected disease research and development: Is innovation under threat? [Internet]. 2011. Available from: http://www.policycures.org/downloads/g-finder_2011.pdf | |
