# Supplementary material for: Global health equity in United Kingdom university research: a landscape of current policies and practices
Source: Health Res Policy Syst. 2016 Oct 10;14:76. doi: 10.1186/s12961-016-0148-6 (PMC5057402; doi:10.1186/s12961-016-0148-6)
Supplement: Additional file 2: — Copy of online survey and FOI request letter. (DOCX 877 kb) [file 12961_2016_148_MOESM2_ESM.docx]

**Online survey (SurveyMonkey)**


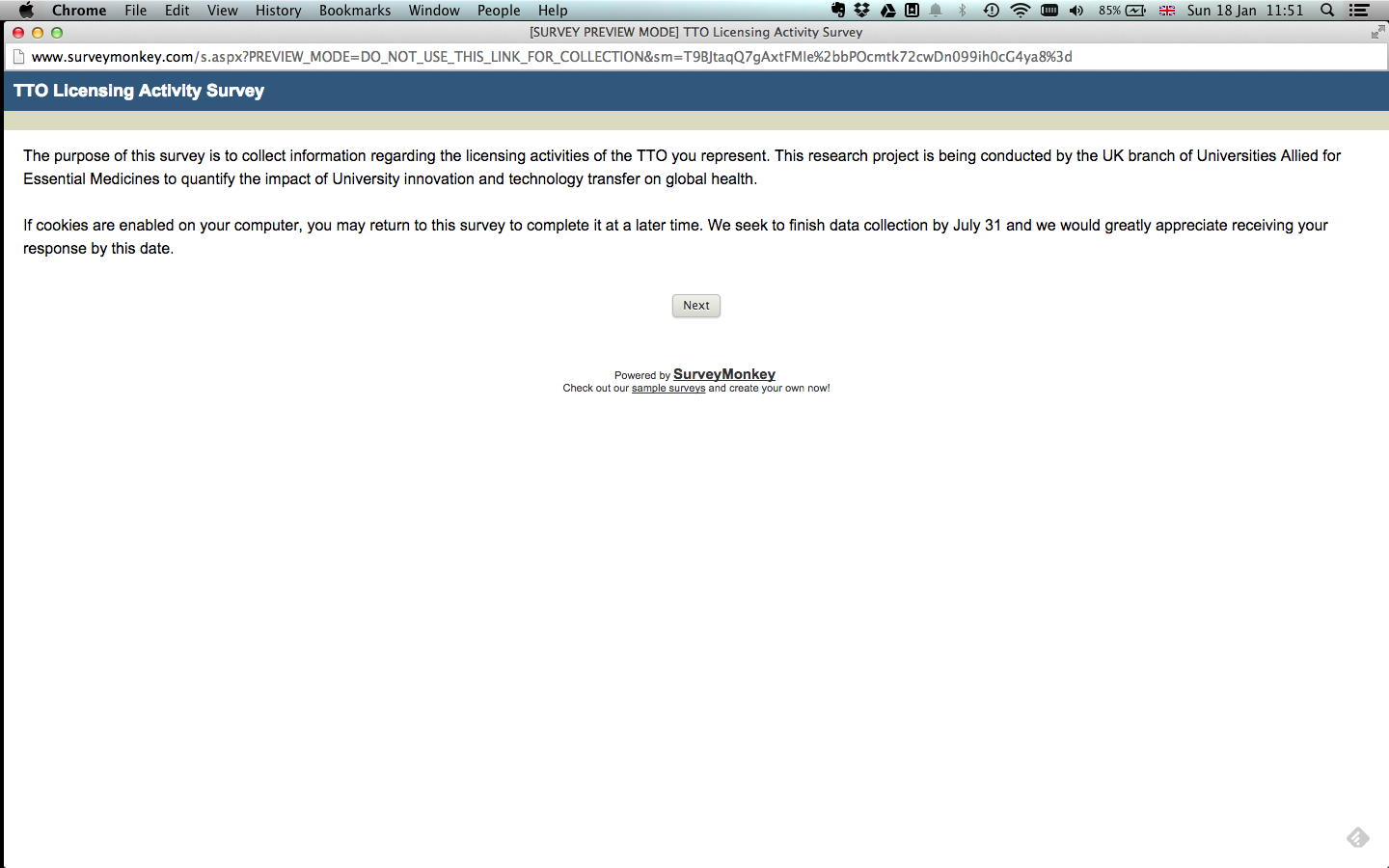


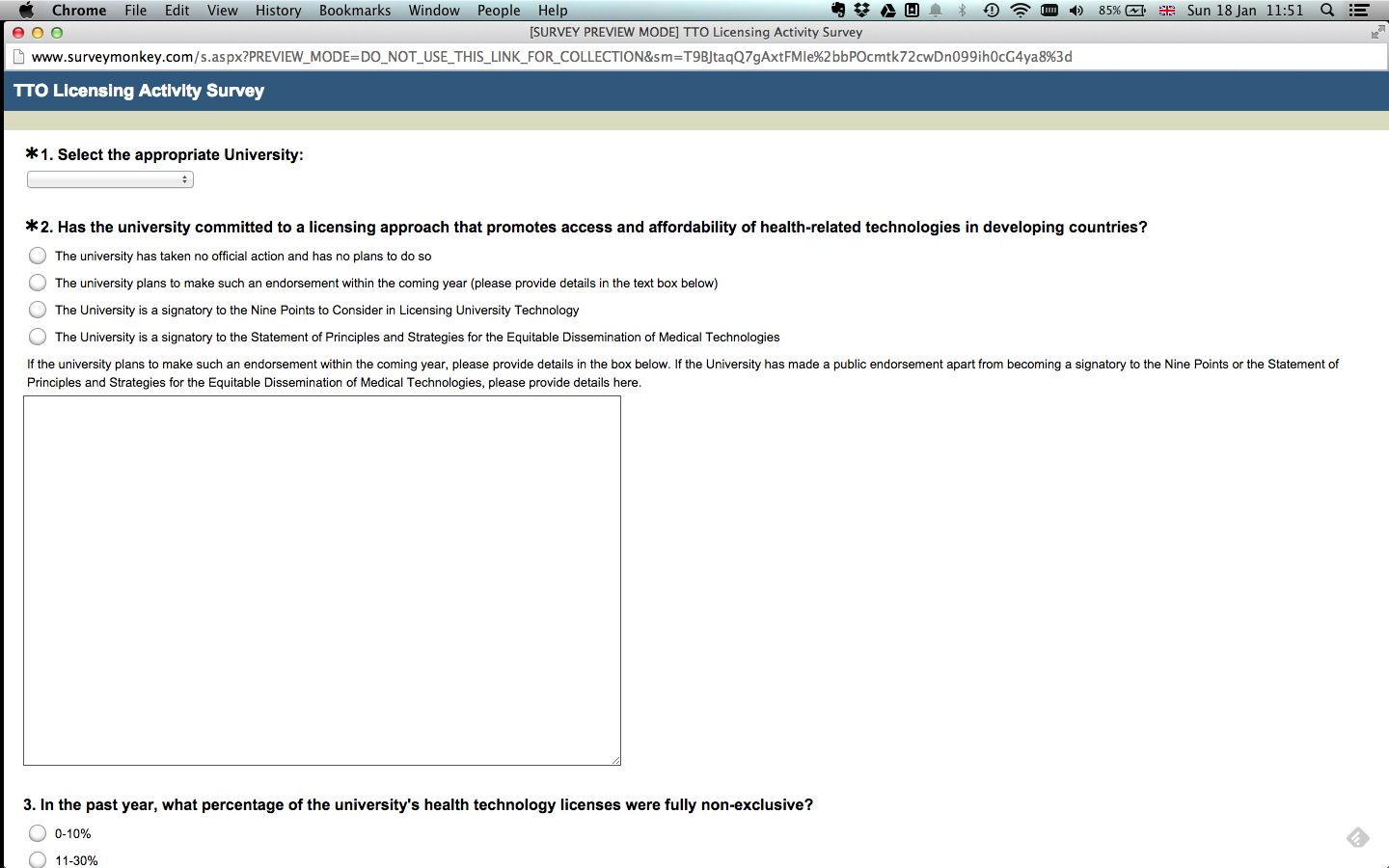


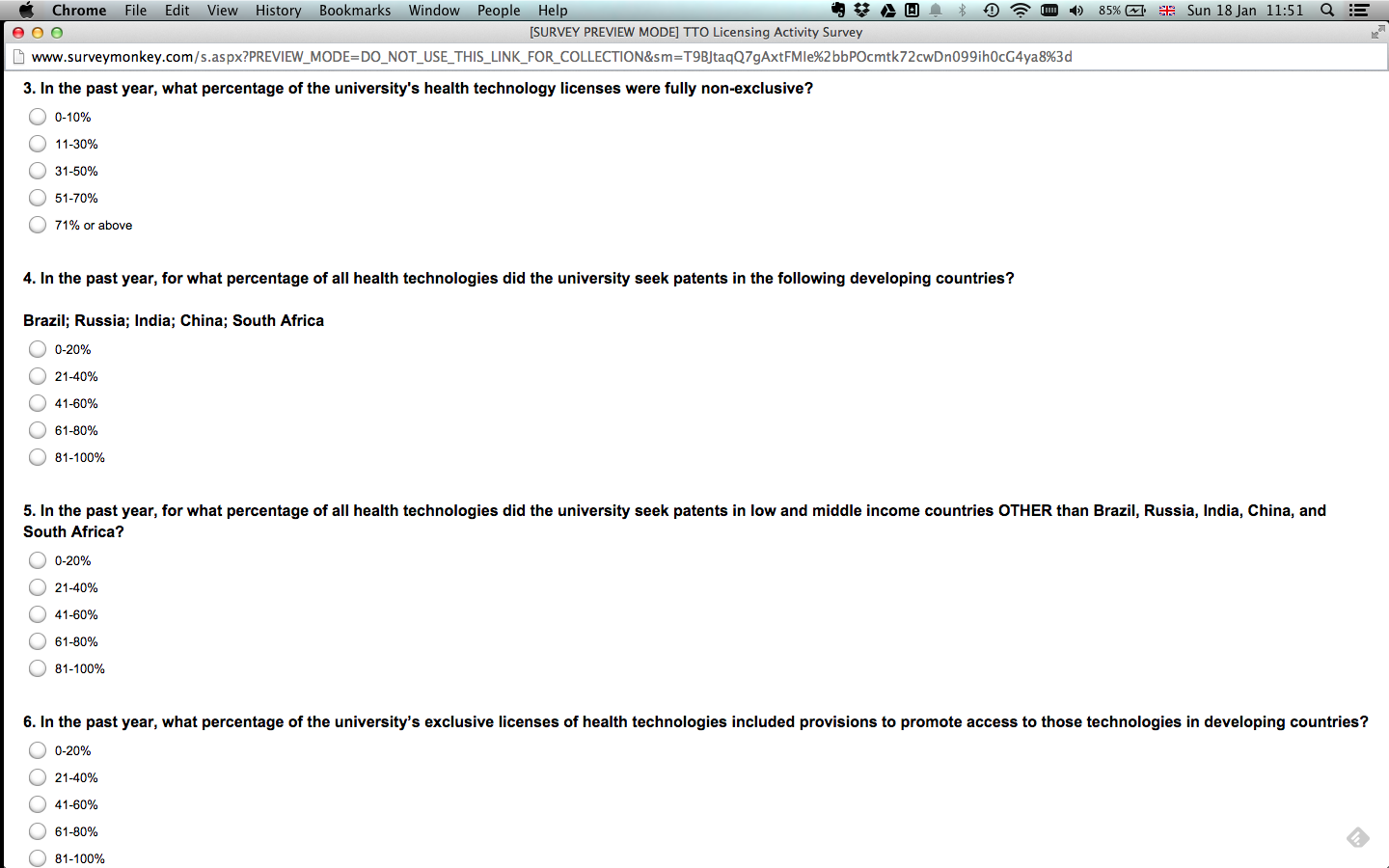


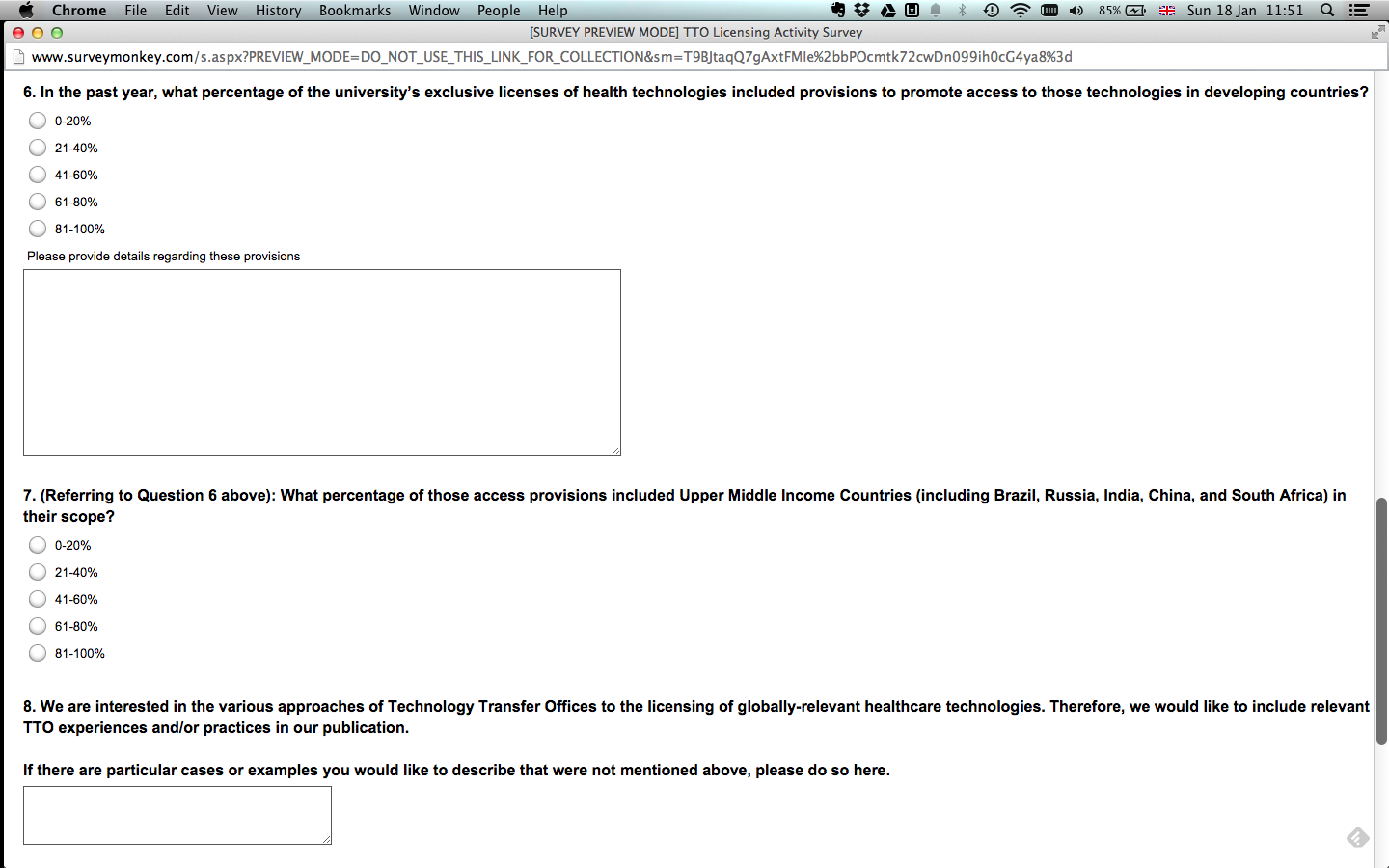


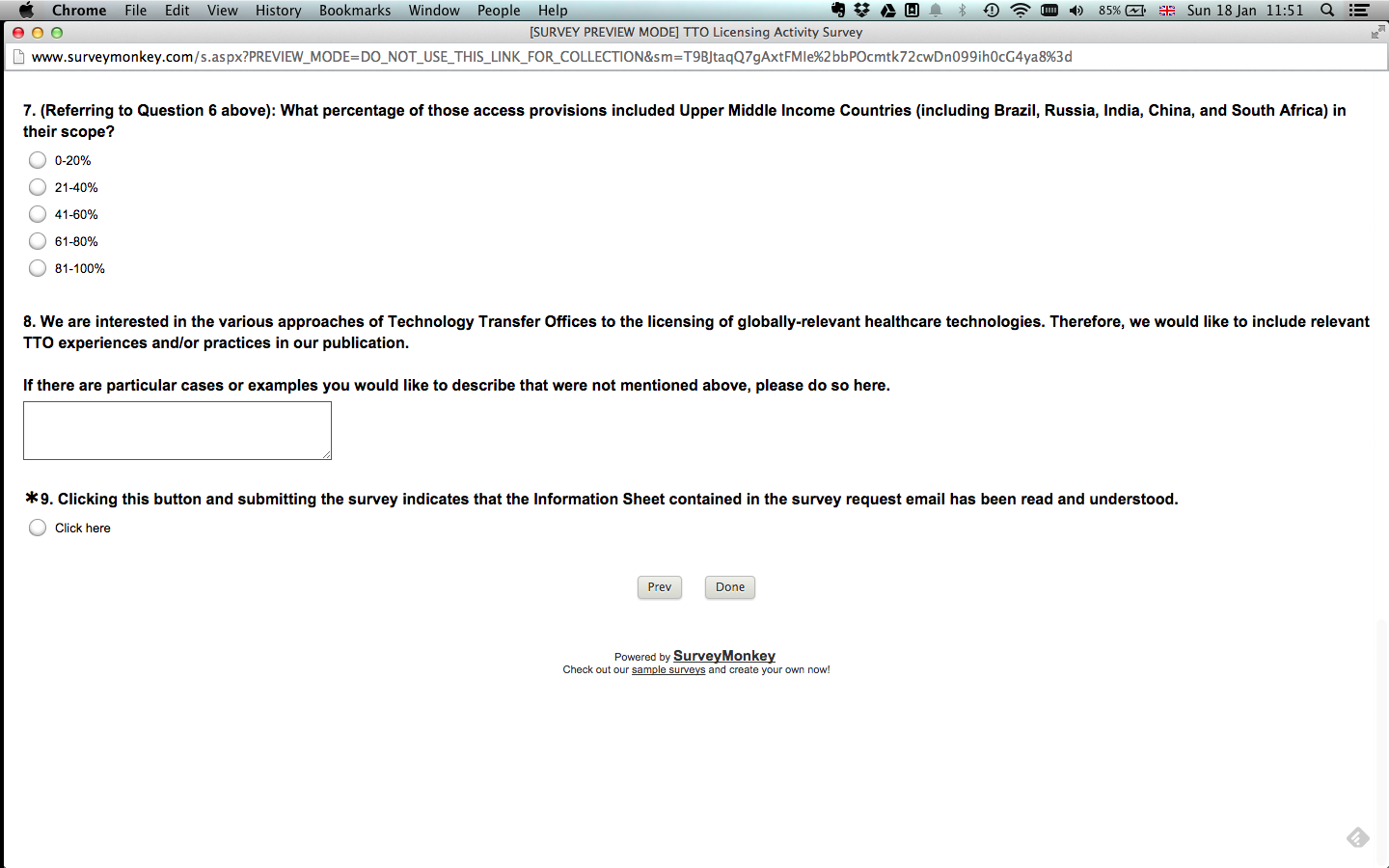


**Request sent under the Freedom of Information Act 2000**

*Email text*

Subject: Freedom of Information request: Healthcare technology licensing research

Summary:

This is a Request for Information under the Freedom of Information Act 2000, made by email on 01/10/2014 at [time].

Please refer to the cover letter document included below.

*Cover letter [attached to email as PDF]*

Dear ______

I write on behalf of a group of researchers investigating healthcare technology licensing by UK universities as part of a project run jointly by Universities Allied for Essential Medicines UK and Medsin UK. These are both student-led organisations with a specific interest in universities' impact on global health. We are interested in the approaches of those responsible for university technology transfer in the licensing of globally-relevant healthcare technologies.

To this end I would like to draw your attention to a series of email and telephone contacts that we initiated beginning July 6 asking for your response to a short 7-question survey on the licensing practices of X University. We note that you have not yet responded to this survey and would like to request information relating to the topics of this survey under the Freedom of Information Act 2000.

The information we are asking for is listed in the attached PDF document. We have also included an Excel spreadsheet into which this information can be recorded.

Finally, if you are able to complete the original survey by the following link, we will not require the information we are requesting under the Act. An Information Sheet is also attached, which provides further details regarding our intended use of the data we collect.

[survey link]

We look forward to receiving your response within 20 working days, that is, by 29/10/2014.

*Information requested [part of ‘Cover letter’ PDF]*

In all points below, “TTO” refers to [specific TTO name] and any other entities that register and license patents deriving from the University’s research.

1. Between 01/08/2013 and 01/08/2014, what number of patents were registered by TTO?
2. Between 01/08/2013 and 01/08/2014, what number of patents pertaining to healthcare and/or biomedical technologies were registered by TTO?
3. Between 01/08/2013 and 01/08/2014, what number of patents pertaining to healthcare and/or biomedical technologies were licensed exclusively to one party?
4. Between 01/08/2013 and 01/08/2014, what percentage of patents pertaining to healthcare and/or biomedical technologies were licensed exclusively to one party? Please report a best estimate if you are unable to report this percentage specifically.
5. Between 01/08/2013 and 01/08/2014, what number of patents pertaining to healthcare and/or biomedical technologies registered by TTO were licensed to private entities?
6. For each patent pertaining to healthcare and/or biomedical technology licensed by TTO between 01/08/2013 and 01/08/2014, please provide the total number of licensees for each patent.
7. Between 01/08/2013 and 01/08/2014, what number of patent licenses pertaining to healthcare and/or biomedical technologies included contractual provisions that exclude parties other than the primary licensee from producing that patented technology?
8. Between 01/08/2013 and 01/08/2014, what percentage of patent licenses included contractual provisions that exclude parties other than the primary licensee from producing that patented technology? Please report a best estimate if you are unable to report this percentage specifically.
9. Between 01/08/2013 and 01/08/2014, for each patent pertaining to healthcare and/or biomedical technologies, please list all countries in which a patent was sought.
10. Between 01/08/2013 and 01/08/2014, for each patent pertaining to healthcare and/or biomedical technologies, please classify the geographical scope of each patent licensing contract made by TTO.
