# Supplementary material for: Global health equity in United Kingdom university research: a landscape of current policies and practices
Source: Health Res Policy Syst. 2016 Oct 10;14:76. doi: 10.1186/s12961-016-0148-6 (PMC5057402; doi:10.1186/s12961-016-0148-6)
Supplement: Additional file 3: — Funding breakdown by funder, and three-year funding trends. (DOCX 80 kb) [file 12961_2016_148_MOESM3_ESM.docx]

| Patent-seeking activity (2012-2013) | | |
| --- | --- | --- |
| University | **% patents sought in BRICS** | **% patents sought in LLMICs*** |
| University of Bristol | 0% | 0% |
| University of Dundee | 0% | 0% |
| University of Nottingham | 0% | 0% |
| University of Southampton | 0% | 100% |
| King's College London | 9.40% | 5.70% |
| Imperial College London | 0-20% | 0-20% |
| University College London | 0-20% | 0-20% |
| University of Aberdeen | 0-20% | 0-20% |
| University of Birmingham | 0-20% | 0-20% |
| University of Glasgow | 0-20% | 0-20% |
| University of Leicester | 0-20% | 0-20% |
| University of Manchester | 0-20% | 0-20% |
| University of Oxford | 0-20% | 0-20% |
| University of Sheffield | 0-20% | 0-20% |
| University of Sussex | 0-20% | 0-20% |
| University of Edinburgh | 21.70% | 0% |
| University of Leeds | 50% | 50% |
| Cardiff University | 100% | 0% |
| Newcastle University | 100% | 0% |
| Queen Mary University | 100% | 0% |
| LSHTM | N/A | N/A |
| University of Liverpool | N/A | N/A |
| University of Reading | N/A | N/A |
| University of Warwick | N/A | N/A |
| University of Cambridge | ** | ** |
| ‘N/A’ indicates no patents were sought during the time period  *Except India.  **Did not provide information requested under the Freedom Of Information Act for this question, citing an exemption. | | |

| Additional tests of correlation | | | | | | | | |
| --- | --- | --- | --- | --- | --- | --- | --- | --- |
|  | **Spearman’s correlation coefficient** | | | | **Number of rows censored in each test (from maximum 25 rows)** | | | |
|  | Proportion of licenses that were non-exclusive | Proportion of licenses that included provisions to promote access in LLMICs | percentage of articles published as free-access | percentage of articles published as CC-BY | Proportion of licenses that were non-exclusive | Proportion of licenses that included provisions to promote access in LLMICs | percentage of articles published as free-access | percentage of articles published as CC-BY |
| Absolute hLLMIC funding | 0.1508 (p=.5503) | -0.0552 (p=.8719) | 0.2623 (p=0.2382) | 0.2831 (p=0.2137) | 7 | 14 | 4 | 4 |
| Absolute ND funding | -0.1099 (p=.6643) | -0.2322 (p=0.4921) | 0.2128 (p=0.3543) | -0.2148 (p=0.3498) | 7 | 14 | 4 | 4 |
| Proportional hLLMIC funding | 0.0355 (p=0.8853) | 0.4308 (p=0.1417) | **0.5877 (p=0.004)** | **0.4910 (p=0.0203)** | 6 | 12 | 3 | 3 |
| Proportional ND funding | -0.0365 (p=0.8787) | 0.0464 (p=0.8803) | 0.292 (p=0.1873) | -0.1916 (p=0.3929) | 5 | 12 | 3 | 3 |
| All correlation tests used Spearman’s rank correlation coefficient. P-values are given in parentheses, with results with p-value < 0.05 in bold. Tests used datasets where rows with outliers and/or non-usable data, for example where universities did not respond to the survey on licensing policies, had been censored. | | | | | | | | |
