# Supplementary material for: Global health equity in United Kingdom university research: a landscape of current policies and practices
Source: Health Res Policy Syst. 2016 Oct 10;14:76. doi: 10.1186/s12961-016-0148-6 (PMC5057402; doi:10.1186/s12961-016-0148-6)

**Total health research funding to 25 study universities, by funder, 2011-2014.**

**
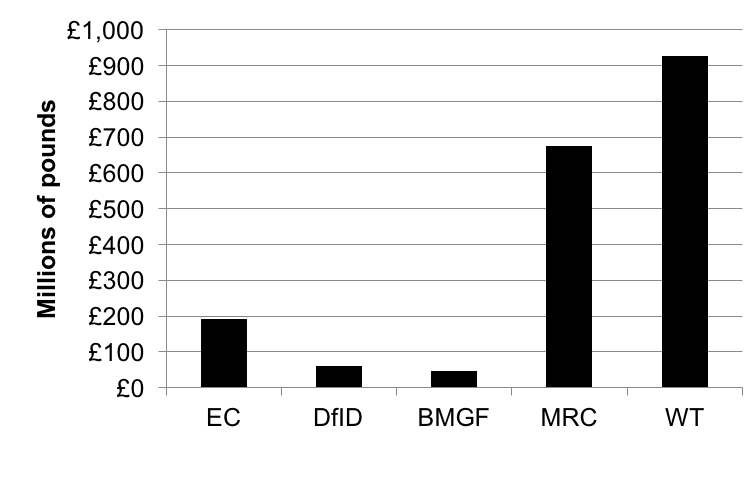
**

**Total funding for research on neglected diseases to 25 study universities, by funder, 2011-2014.**

**
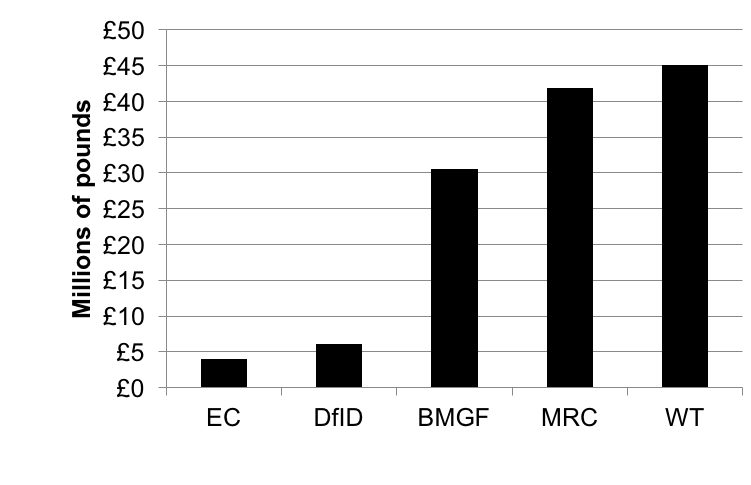
**

**Total funding for research on health in low- and lower-middle-income countries to 25 study universities, by funder, 2011-2014.**

**
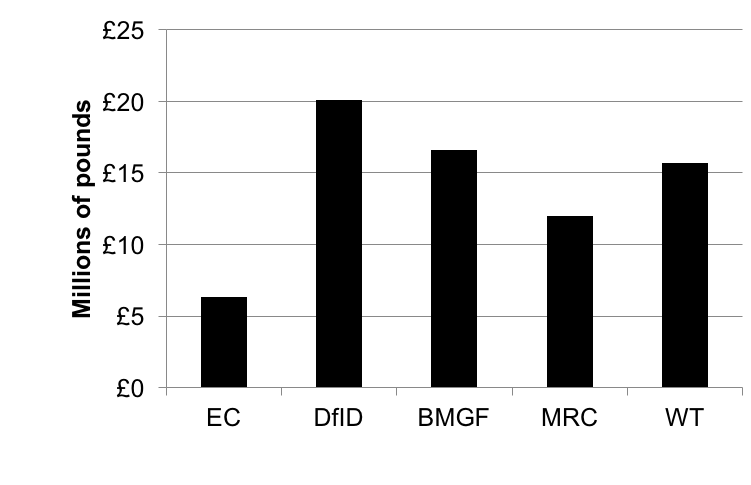
**

EC, European Commission; DfID, (UK) Department for International Development; BMGF, Bill and Melinda Gates Foundation; MRC, Medical Research Council; WT, Wellcome Trust.

**Total health research funding, by year, 2011-2014.**


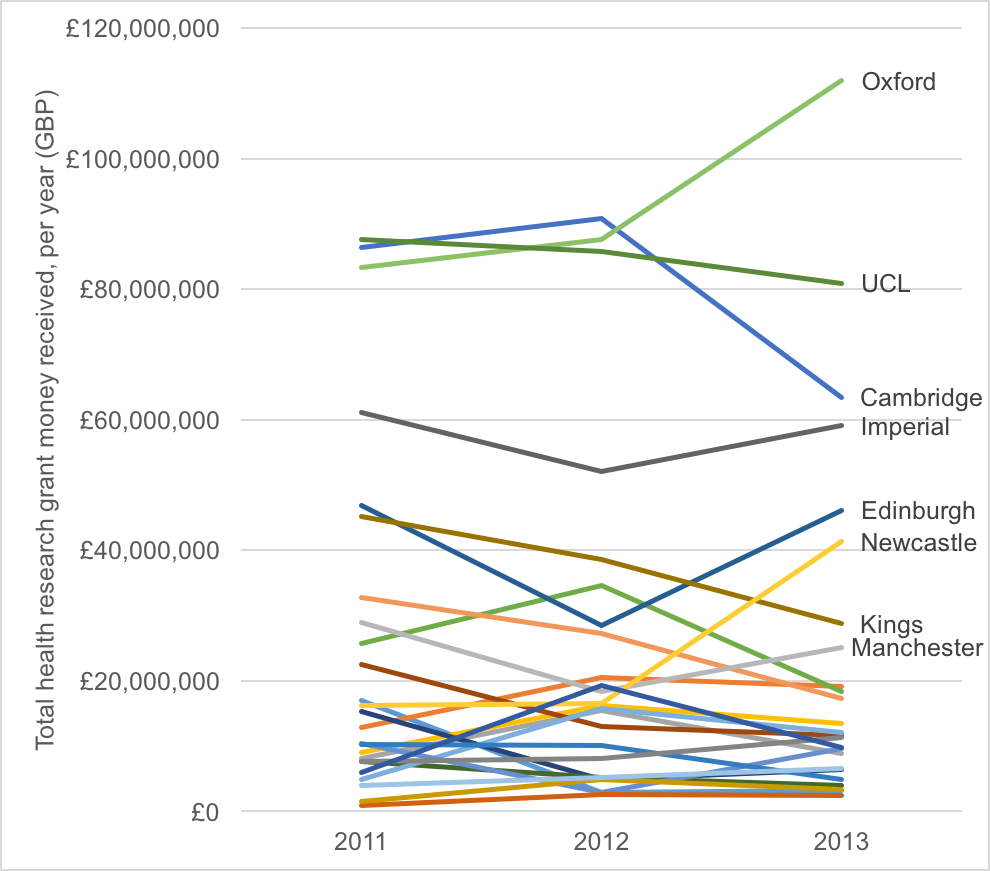


**Total health funding for research on neglected diseases, by year, 2011-2014.**

**
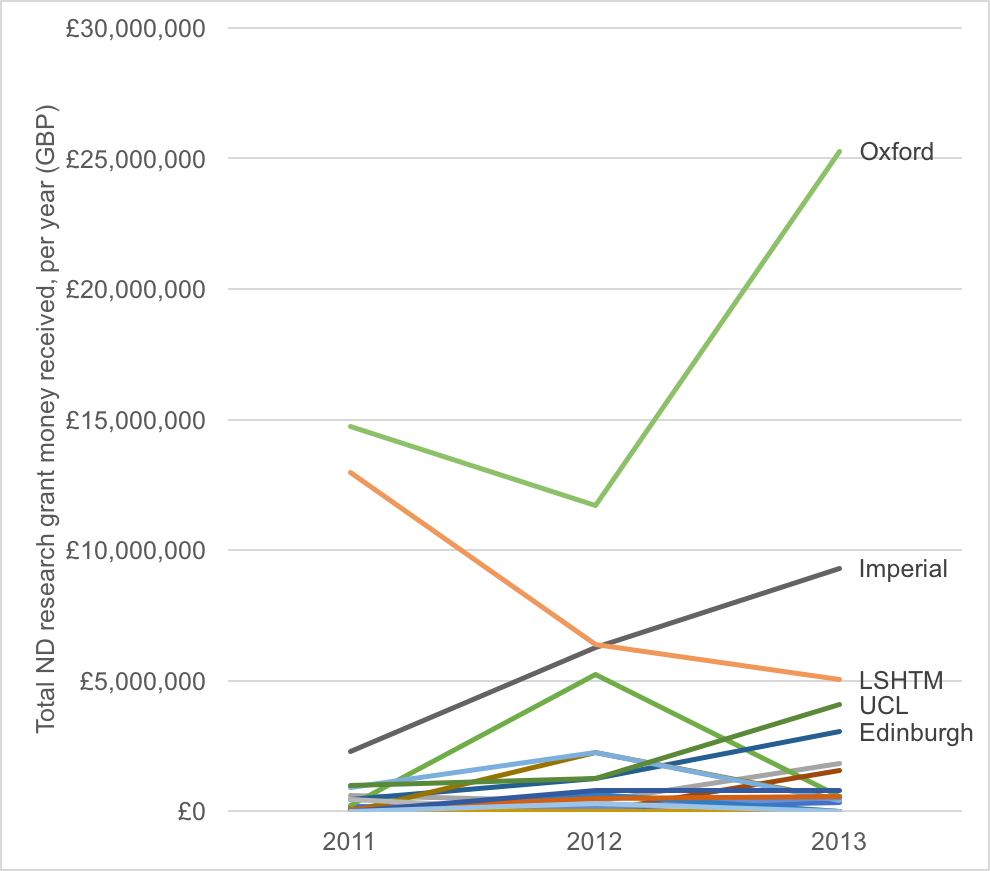
**

**Total health funding for research on health in low- and lower-middle-income countries, by year, 2011-2014.**


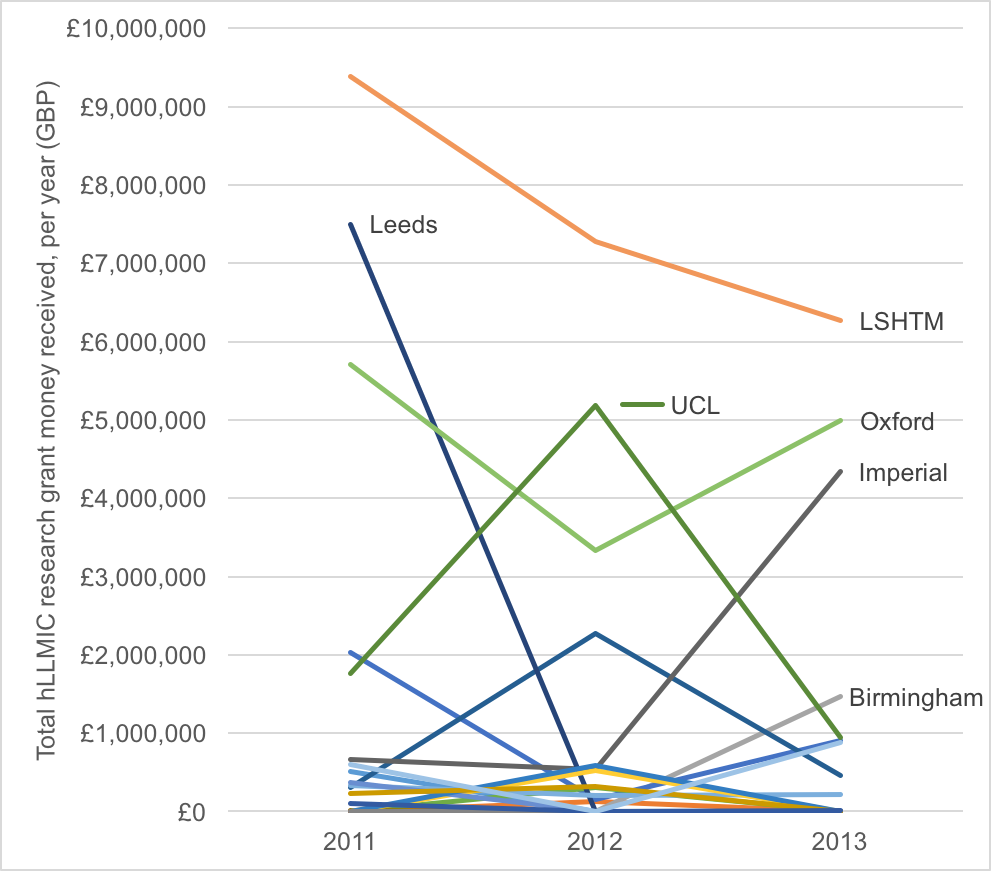


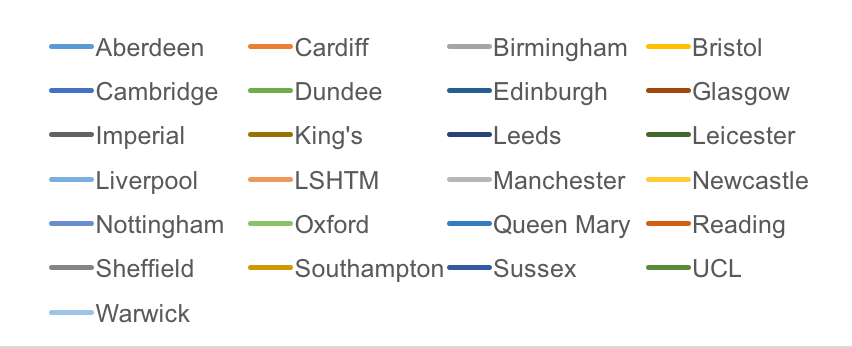

Supplement: Additional file 4: — Patenting activity in BRICS countries and LLMICs by university, and additional inter-metric correlation statistics. (DOCX 594 kb) [file 12961_2016_148_MOESM4_ESM.docx]
